# Feces Disposal and Fly Prevention

**Published:** 1918-10

**Authors:** 


### FECES DISPOSAL AND FLY PREVENTION

It may be stated as a fact, which no sanitarian will question, that if all the excreta in the neighborhood of soldiers could be destroyed, and none of their food contaminated by infected feces, typhoid and paratyphoid fevers and the various types of dysenteries, which cause so many fighting men to be in hospitals when they are needed on the firing line, would be eliminated from army life. Therefore the solution of the typhoid fever and dysentery problem would seem to resolve itself into educating soldiers first to dispose of their own feces, so that it cannot get into the mouths of others; and then to teach them how to keep other peoples' feces from getting into their own food and drink. This may not be a very elegant exposition of this great sanitary problem of the army, but it is a plain statement of the situation in the case; and soldiers should have unvarnished facts stated to them in order that they may be impressed sufficiently with the necessity for doing their part in the proper disposal of feces. Apparently this is a simple problem, and one that ought not to exist among civilized people; but the difficulties of feces disposal and the prevention of food contamination in a rapidly moving army are very great.

At the September meeting of the Research Society, a session was devoted to a discussion of "Feces Disposal and Fly Prevention". Many interesting and instructive facts were brought out by British, French and American medical officers. It was generally agreed by them that incineration of feces is the surest method of disposal, but it is difficult to get fuel in France, and the smoke from incinerators may give the enemy information regarding the location of troops. Incinerators are also difficult of transportation, and are therefore not adapted for use in rapid advances such as have been made recently by the Allies. Under these circumstances, the best method of feces disposal is to bury it as Moses advised 4000 years ago.

### Sanitation in Areas Evacuated by the Germans.

With the Germans retreating as they have been recently, and with the likelihood of further "strategic withdrawals from untenable positions" by them, the Allies have not only to look out for the disposal of the excreta of their own soldiers, but a larger problem is the sanitation of the places evacuated by the enemy. From the unspeakable conditions left by the Germans in the Marne area, as described by one of the officers, and judging from the fact that an epidemic of dysentery among the Allied troops followed the occupation of territory that had been occupied by the enemy, it seems that German feces is more to be feared than Krupp guns. Someone has suggested that "the reason the Boche are called Germans is because they are so full of " germs ". At any rate sanitary officers say that it is more difficult to " clean up " after the Germans, than it is to " clean them up " in battle.

Fly prevention is a serious problem, the solution of which, as brought out in the discussion before the Research Society, is the proper disposal of the excreta and dead bodies of animals, for in this manner the principal breeding places for flies are suppressed. This measure involves the co-operation of the civil population, which is at times difficult to obtain. One of the speakers said that the big blue flies that breed in decaying flesh of animals, and sometimes even in that of human beings in battle areas, are not so dangerous because that variety contaminates only meat, which is sterilized in cooking ; but the house fly that breeds in manure is the one that goes from the latrines to the mess table and kitchen, and deposits bits of human feces on bread, sugar, and other food that will not be cooked before it is eaten. A point of importance that was stressed is that while every effort should be put forth to prevent fly breeding near soldiers' camps, the proper disposal of feces is of the greatest importance, because flies get the bacilli of dysentery, typhoid, and paratyphoid from the feces of infected human beings.

EDUCATION ON PERSONAL HYGIENE AND ARMY DISCIPLINE NEEDED.

As brought out by several of the speakers, particularly by Colonel Gressinger, education and discipline of the soldiers regarding feces disposal and food contamination is the only method of preventing the enteric group of infections. Every soldier in the army should be so well trained and disciplined that under no circumstances, not even in the exhaustion following a battle, will he be guilty of soil pollution.

After all, army sanitation is a rural problem and should be attacked as it has been in some of the country communities in the United States, where every inhabitant has been taught the dangers of soil pollution, and where sanitary methods of feces disposal have been installed in every home. It therefore would seem that the liaison officer with the civil authorities is an important official, and that through him the co-operation of the civil population might be obtained.

While it is true that diseases due to food and water contamination are less prevalent in winter than in summer, cold does not destroy the organisms causing them. Soil pollution in the winter means that with the thawing of the snow, and the spring rains, streams will be contaminated and serious outbreaks will occur in warm weather. It is therefore important that the practical methods of feces disposal and fly prevention, as presented to the Research Society, the report of which is published in *War Medicine,* should be carried into effect in each unit of every division of the American Expeditionary Forces at the earliest possible time. It is up to the division and regimental surgeons to get the active co-operation of the line officers in a campaign of education on sanitary matters, which will extend throughout the army. Through them they can teach every soldier the dangers of soil pollution, and that it is as much his duty to protect himself and his comrades from the enemies behind and within the lines, that lurk in contaminated food and drink, as it is for him to "go over the top" after the foes that he can see.

